# Diversified pulse generation from frequency shifted feedback Tm-doped fibre lasers

**DOI:** 10.1038/srep26431

**Published:** 2016-05-19

**Authors:** He Chen, Sheng-Ping Chen, Zong-Fu Jiang, Jing Hou

**Affiliations:** 1 College of Optoelectronic Science and Engineering, National University of Defense Technology, Changsha 410073, China

## Abstract

Pulsed fibre lasers operating in the eye-safe 2 μm spectral region have numerous potential applications in areas such as remote sensing, medicine, mid-infrared frequency conversion, and free-space communication. Here, for the first time, we demonstrate versatile 2 μm ps-ns pulses generation from Tm-based fibre lasers based on frequency shifted feedback and provide a comprehensive report of their special behaviors. The lasers are featured with elegant construction and the unparalleled capacity of generating versatile pulses. The self-starting mode-locking is initiated by an intra-cavity acousto-optical frequency shifter. Diversified mode-locked pulse dynamics were observed by altering the pump power, intra-cavity polarization state and cavity structure, including as short as 8 ps single pulse sequence, pulse bundle state and up to 12 nJ, 3 ns nanosecond rectangular pulse. A reflective nonlinear optical loop mirror was introduced to successfully shorten the pulses from 24 ps to 8 ps. Beside the mode-locking operation, flexible Q-switching and Q-switched mode-locking operation can also be readily achieved in the same cavity. Up to 78 μJ high energy nanosecond pulse can be generated in this regime. Several intriguing pulse dynamics are characterized and discussed.

In recent years, continuous wave as well as pulsed fibre lasers operating in the eye-safe 2 μm spectral region have attracted extensive attention owing to their numerous potential applications in areas such as remote sensing, medicine, mid-infrared frequency conversion, supercontinuum generation, and free-space communication. Various technologies have been explored in this region to produce pulses with diverse characteristics for the applications of different requirements, such as mode-locking, Q-switching and gain-switching. Generally, mode-locking is used to generate fs-ps ultrafast pulses and Q-switching and gain-switching are exploited to produce ns-μs high energy pulses. Currently, most passive mode-locking techniques exploited in the 2 μm region are based on saturable absorbers, which can be classified into two categories: real saturable absorbers and artificial saturable absorbers. The former is based on materials with saturable absorbing characteristics, such as traditional SESAM, carbon nanotube, Graphene, and various emerging low-dimension materials^1^,^2^,^3^,^4^,^5^,^6^,^7^. The later mainly relies on the nonlinear optical effects, including nonlinear polarization rotation (NPR) and nonlinear optical loop mirror (NOLM). Although saturable absorber can also be utilized to initiate passive Q-switching operation, the performances and temporal tunabilities of passively Q-switched lasers are usually no match for actively Q-switched ones.

Beside the common mode locking techniques mentioned above, there is another intriguing technique can be exploited to initial mode-locking, which is called frequency shifted feedback (FSF). Pulse formation in FSF lasers was firstly reported by Kowalski *et al.*^8^, and has previously been studied in Er-doped and Yb-doped fibre lasers^9^,^10^,^11^,^12^,^13^,^14^,^15^,^16^,^17^,^18^,^19^,^20^,^21^. The key component of the FSF mode locked fibre laser is an intra-cavity frequency shifter, which is typically also an unmodulated acousto-optical modulator (AOM), which is in charge of transferring energy among different laser resonant frequencies. The formation of the mode-locked pulses in a FSF laser can be attributed to the interaction between the frequency shifter, the effect of self-phase modulation, and the spectral filter. Complete descriptions of FSF lasers can be found in^22^,^23^. Beside the pure FSF mode-locked fibre lasers, there were also mode-locked fibre lasers based on the combined action of FSF and nonlinear saturable absorbing effect, such as NPR and SESAM, which could generate ultrafast sub-ps pulses^14^,^15^,^16^,^19^.

The FSF technique has been proved to be a competitive alternative to those saturable-absorber-based mode-locking techniques. FSF lasers are usually compactly constructed, polarization insensitive, self-starting and environmentally stable^12^,^18^. It can also be used to develop all-fibre single polarization mode-locked sources if an all-fibre polarization maintaining frequency shifter is employed. Besides, AOM usually has much higher power handling capacity than most saturable absorbers, which makes FSF an ideal scheme to generate high power pulses directly from the oscillator. As high as 8 W average power ps pulses and sub-picosecond pulses with 40 kW peak power had been obtained with this technique^18^,^20^. Another distinct advantage of FSF laser is that we can obtain controllable Q-switching and Q-switched mode-locking (besides normal continuous wave mode-locking) by modulating the intra-cavity AOM (which is employed as the frequency shifter in mode-locking regime). Thus, both of the fs-ps mode-locked pulses and ns-μs high energy pulses can be generated in the same laser cavity, which largely extend the scope of the laser’s possible applications.

FSF had been adopted in fibre gain media based on ytterbium and erbium to generate optical pulses, however, there is no report on the availability and the peculiarity of FSF in other gain media and operating wavelengths. To expand the application range of the FSF mode locking technique and help contribute to the knowledge of mode-locked laser dynamics, in this manuscript, we demonstrate the first implementation of Tm-based FSF laser and present a comprehensive characterization of its special output pulse dynamics. Two novel cavity constructions are proposed and adopted to improve the laser’s structural simplicity, robustness and performances. Versatile mode-locking pulse dynamics and widely tunable Q-switching and Q-switched mode-locking operation were experimentally manifested and comprehensively analysed.

## Results and Discussion

**Figure 1:**
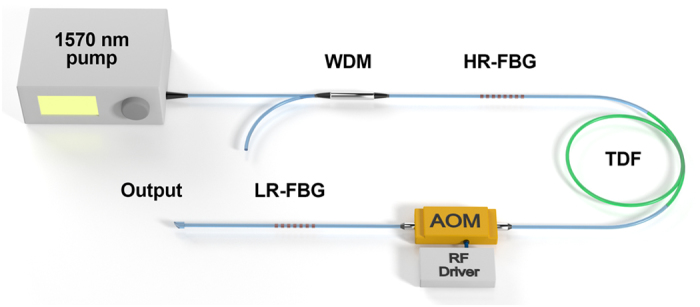
The schematic diagram of the FBG-pair-based FSF mode-locked fibre laser. TDF, thulium-doped fibre; WDM, wavelength division multiplexer; HR-FBG, high-reflectivity fibre Bragg grating; LR-FBG, low-reflectivity fibre Bragg grating; AOM, acousto-optical modulator; RF driver, radio frequency driver.

In the experiment, two structures were adopted to achieve mode locking based on FSF in Tm-doped fibre and 2 μm region. Both of them were based on the linear Fabry-Perot cavity structure, which included a fiberized AOM as the frequency shifter. The difference was that the former one was formed with a pair of fibre Bragg gratings (FBGs), while the later one was formed with an FBG and an NOLM as the cavity mirror and the additional saturable absorber. The schematic diagram of the FBG-pair-based structure is shown in Fig. 1. The FBG pair are centred at 1980 nm. The high-reflectivity (HR) FBG has a reflectivity of 99.1% and a 3 dB bandwidth of 1.3 nm. The low-reflectivity (LR) FBG has a reflectivity of 20% and a 3 dB bandwidth of 0.8 nm. A section of 2-meters-long single mode 9/125 Tm-doped fibre was employed as the gain medium. The core absorption coefficient is around 10 dB/m at 1570 nm, and the numerical aperture is 0.15. The pump light came from a 1.3 W 1570 nm home-made fibre laser. A 2000/1570 nm WDM was inserted between the pump source and the HR-FBG to prevent 2 μm backward light. The AOM frequency shifter was inserted between the gain fibre and the LR-FBG, which had frequency shift of 80 MHz and insertion loss of 2 dB. A matched 80 MHz radio frequency (RF) driver was employed to activate the AOM. The total cavity length was tailored to be ~6.45 m, corresponding to the fundamental cavity frequency of ~16 MHz (1/5 of the AOM’s shift frequency). At the wavelength of 1980 nm, the cavity is anomalously dispersive, and the total cavity dispersion was estimated to be ~−0.98 ps^2^ at the wavelength of 1980 nm. It is a notable feature that this laser is all-fibre-integrated and compactly constructed, which is favourable in many practical applications.

**Figure 2:**
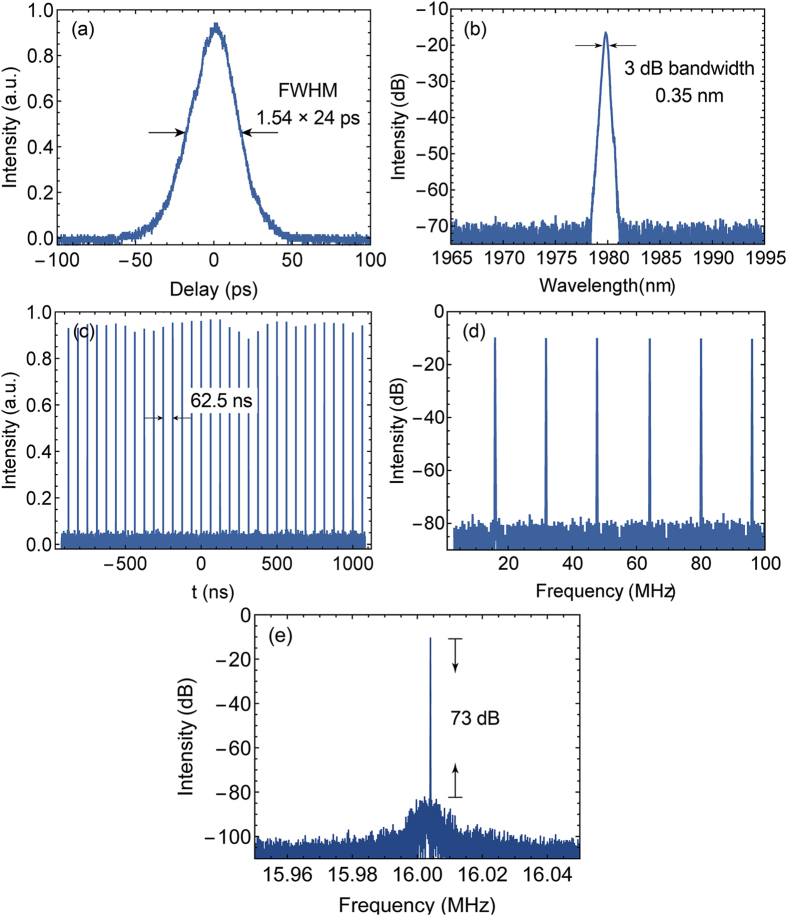
(**a**) The autocorrelator trace; (**b**) The optical spectrum; (**c**) The oscilloscope trace; (**d**) The RF spectrum of the output pulse under the pump power of 220 mW; (**e**) The RF spectrum with the span of 100 kHz and resolution bandwidth of 10 Hz.

Stable single pulse mode locking could be instantly self-started from the continuous wave (CW) regime as long as the pump power was increased to around 200 mW. Single pulse operation can be maintained until the pump power was increased above 240 mW. Then the output pulse split to amplitude-even pulse bundle. Figure 2 depicts the measured temporal and spectral characteristics of the FSF mode-locked laser in the single pulse regime at the pump power of 220 mW. The output power of 7.4 mW was obtained at this pump power. Figure 2(a) shows the measured autocorrelation trace with a full width at half maximum (FWHM) of 37 ps. If a sech^2^ pulse profile was assumed, the pulse width was measured to be 24 ps. The spectrum of the output pulse is shown in Fig. 2(b), which is centred at 1980 nm with a 3 dB bandwidth of 0.35 nm. Then the calculated time-bandwidth product of output pulse is 0.65, which is larger than the theoretical value for transform-limited sech^2^ pulse indicating a small amount of negative dispersion in laser pulses. Figure 2(c) plots the measured pulse train with a time interval of ~62.5 ns between each pulses corresponding to a pulse repetition rate of 16 MHz, which corresponded to the estimated cavity round trip. The radio frequency spectrum measured in the span of 100 MHz (resolution bandwidth 100 Hz) and the span of 100 kHz (resolution bandwidth 10 Hz) are shown in Fig. 2(d,e), respectively. The signal-to-noise ratio of the RF spectrum is measured to be as high as 73 dB, indicating a high temporal stability^24^. The measured autocorrelator trace and the RF spectrum manifested the single pulse operation. With the 16 MHz repetition rate and 7.4 mW average output power, the pulse energy was calculated to be 0.46 nJ. To investigate the effect of the cavity length on the mode-locking operation. We also shortened the cavity length to ~6.3 m to make the cavity frequency mismatch the frequency shift of the AOM. Mode-locking behaviours were also observed. The differences were the pump threshold for the mode locking was around 310 mW, which was higher than the case with the matched cavity length, and the pulse train was less stable.

**Figure 3:**
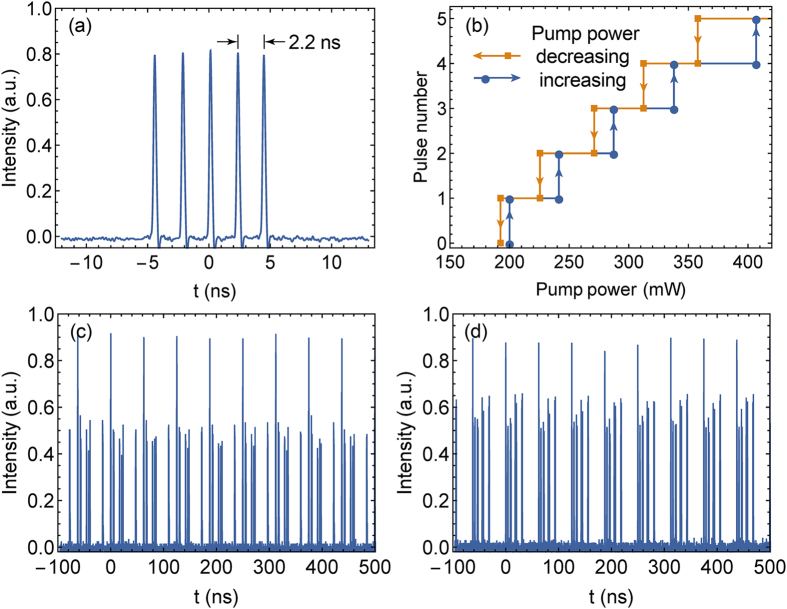
(**a**) The oscilloscope trace of the 5-pulse-bundle; (**b**) The variation of the number of the sub-pulses versus the increasing and decreasing pump power; The oscilloscope trace of the output pulse train under the pump power of (**c**) 0.6 W and (**d**) 0.9 W.

As mentioned above, the output pulses were transformed from the single pulse state to pulse bundle state upon the pump power was increased to around 240 mW, which was caused by the peak power clamping effect^25^,^26^. With the increasing pump power, the number of the pulses in single pulse bundle increased accordingly, while the repetition rate of the pulse bundle remained at the fundamental frequency 16 MHz. Up to 5-pulse-bundle was formed with the pump power of 410 mW. In this case, the output average power reached 48 mW, and the corresponding energy of the pulse bundle was calculated to be 3 nJ. The waveform of the 5-pulse-bundle measured by a 1.5 GHz oscilloscope is shown in Fig. 3(a). The inner pulse width was measured to be ~200 ps, which was limited by the bandwidth of the oscilloscope. The pulse intervals between the inner pulses were not quite stable, dithering around 2.2 ns with a timing jitter of ~200 ps.

Hysteresis phenomenon of the pulse number of the pulse bundle versus the pump power was observed, as shown in Fig. 3(b). The pulse numbers in Fig. 3(b) correspond to the pulse bundle states with 1–5 sub-pulses. As we can see, the n-pulse-bundle transformed to n + 1-pulse–bundle when the pump power was increased to a certain threshold P_n_. However, when the pump power was decreased gradually to the threshold P_n_, the state of (n + 1)-pulse–bundle maintained until the pump power dropped below 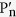, and 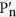 < P_n_. This hysteresis phenomenon continued and faded until the pump power fall below the mode-locking threshold. This hysteresis phenomenon had been demonstrated in many other passively mode-locked fibre lasers, including Er-doped frequency shifted feedback lasers^21^, and they were based on the same mechanism with the hysteresis phenomenon of the harmonic mode locking order versus the pump power in the harmonically mode-locked fibre laser.

As we continued to increase the pump power, more sub-pulses emerged, but the sub-pulses went unstable and drifting. Both of the sub-pulse intensity and the interval between the sub-pulses became fluctuating. Figure 3(c,d) show the oscilloscope trace of the output pulse train under the pump power of 0.6 W and 0.9 W respectively. In a single pulse bundle, there was a major sub-pulse surrounded with several minor sub-pulses. The pulse number and the pattern of the pulse bundle vary with the change of the pump power. Even with the pump power and the cavity firmly fixed, the intensities and the positions of the minor sub-pulses frequently fluctuated and randomly drifted between the adjacent major pulses. This multi-pulse phenomenon resembles the so-called soliton rain regime, which was firstly proposed and investigated in NPR-based Er-doped fibre lasers^27^,^28^, and also observed in Graphene-oxide mode-locked Yb-doped fibre laser^29^ and NOLM-based Tm-doped fibre lasers^30^. Soliton rain consists of three key parts: the major condensed soliton phase (which corresponds to the major pulse in Fig. 3(c,d)), the drifting minor pulses, and the noisy background. However, in our case, the noisy background wasn’t observed, and the drifting of the minor pulses seems to be random, which are inconsistent with the features of the soliton rain^27^,^28^,^30^. Hence, despite the similarities, we currently cannot categorize this regime to soliton rain before more specific investigation is conducted.

**Figure 4:**
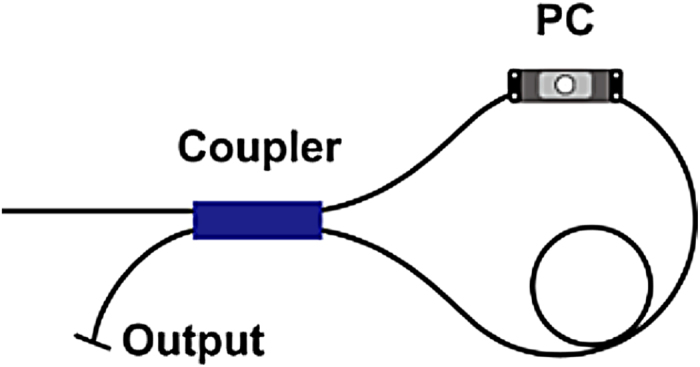
The schematic diagram of the nonlinear optical loop mirror. PC: polarization controller.

The duration of the pulse generated from FBG-pair-based structure is relatively long. For the purpose of further narrowing down the pulse width, we replaced the narrow bandwidth (0.8 nm) LR-FBG with an NOLM based a fused optical coupler. The coupler owns a coupling ratio of 10:90, and the loop length is 2 m. A polarization controller (PC) was inserted in the loop to control the linear phase shift that allows biasing the curve for increased reflection/transmission near zero power, and control its saturable absorber characteristics. As the NOLM has intensity-related nonlinear reflection/transmission characteristics and a reflectivity of 36% at the maximum reflective window, it can simultaneously act as the output coupling mirror and an artificial fast saturable absorber. The NOLM was adopted in the cavity to shorten the output pulse duration. On one hand, it took the advantage of its nonlinear saturable absorbing characteristics. On the other hand, as a reflective mirror, the bandwidth of the NOLM is much larger than the bandwidth of the FBG, which might eliminate the restrictions of FBG’s bandwidth on the output pulse spectral bandwidth, then help to shorten the pulse duration. The schematic diagram of the used NOLM is depicted in Fig. 4. Although the cavity structure was altered, the cavity length was still tailored to the same to make the inverse round-trip of the laser (16 MHz) to be 1/5 of the AOM’s shift frequency (80 MHz). The total dispersion of the laser cavity with NOLM was also estimated to be −0.98 ps^2^/nm. To be mentioned, unlike the commonly used scheme of combining FSF with complex NPR^14^,^16^, for the first time, we introduce a simplified all-fibre NOLM into a linear cavity FSF laser to control the mode-locking behaviours.

**Figure 5:**
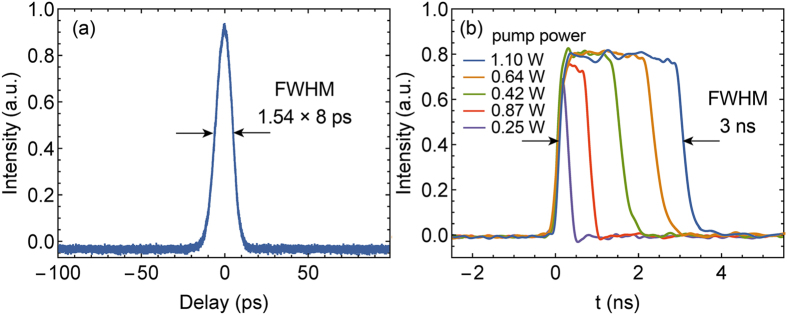
(**a**) The autocorrelator trace of the picosecond output pulse under the pump power of 220 mW; (**b**) The oscilloscope traces of nanosecond rectangular pulses under different pump powers.

With the aid of the NOLM-based fast saturable absorber and the replacement of the bandwidth-limiting LR-FBG, the duration of the pulse from the new laser cavity was greatly shortened. Under the pump power of 220 mW, with proper polarization controlling, the output pulse duration was as short as 8 ps. Figure 5(a) exhibits the measured autocorrelation trace with a FWHM of 12 ps. Assuming a sech^2^ pulse profile, the pulse width is measured to be 8 ps. The corresponding 3 dB spectral bandwidth broadened to 0.8 nm, and then the time-bandwidth product of the output pulse is calculated to be 0.49. The compressed pulse duration manifested that the employment of the NOLM actually played an important role in formation of mode-locked pulses.

Interestingly, in this laser construction, with proper polarization controlling and adequate pump power, the laser could produce widely tunable nanosecond rectangular pulses. The pulse duration and the pulse energy increased almost linearly with the pump power, while the peak power stayed almost unchanged. With the pump power increased from 0.25 W to 1.1 W, the output pulse duration almost linearly increased from <200 ps to 3 ns, as depicted in Fig. 5(b). Under the pump power of 1.1 W, the average power of the rectangular pulse reached 198 mW, and the corresponding pulse energy is calculated to be 12.3 nJ. Limited by the measurement bandwidth of the oscilloscope, we cannot observe the possible fine structure in the rectangular waveforms. However, the autocorrelator trace of these rectangular pulse shows a ~10 ps minor peak on a broad pedestal (exceeding the scanning range), which indicates that the pulses are very likely not unitary pulses but formed with ~10 ps minor pulses. This kind of high energy rectangular pulse cluster has been observed in other mode-locked fibre lasers^31^,^32^. While, to the best of our knowledge, this is the first demonstration of duration-tunable rectangular pulse cluster in a FSF laser.

The various mode-locking regimes demonstrated above were based on the frequency shifting function of the AOM, where the modulator was not actually modulated. When the AOM is employed as the intra-cavity intensity modulator to switch the Q-factor of the cavity (i.e. the cavity loss), flexible Q-switching and Q-switched mode-locking operation can also be facilely achieved. In this case, the FBG-pair-based laser cavity was adopted and we used an electrical pulse generator to modulate the power of the RF driver which is linearly proportional to the insertion loss of the AOM. The repetition rate and duty cycle (laser on time/laser off time) of the modulation signal can be tuned in wide ranges, which will directly affect the characteristics of the Q-switched output pulses. Whether the laser operates in the Q-switching or the mode-locking operation state mainly depends on whether the AOM is periodically modulated or not, i.e. the AOM acts as a Q-switcher or a frequency shifter. In the mode-locking state, the picosecond pulses were generated as a result of the interaction among bandwidth-limited amplification, frequency shifting and self-phase modulation. While in the Q-switching state, the nanosecond pulses were generated as a result of periodically switching the Q-factor of the cavity.

**Figure 6:**
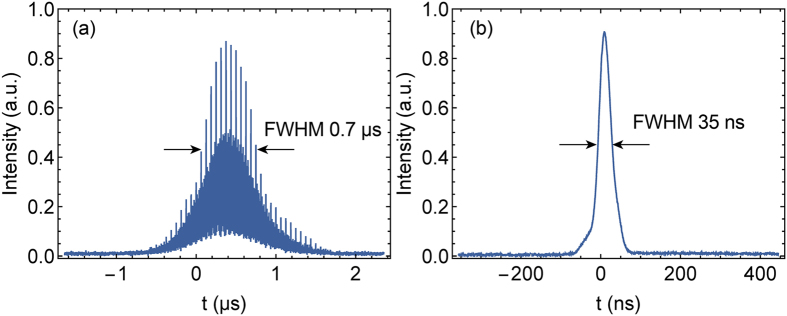
The oscilloscope traces of the output pulse waveforms (**a**) in Q-switched mode locking regime and (**b**) short pulse Q-switched regime.

Under the modulation signal of large duty cycle (typically >50%, dependent on the repetition rate), the laser can produce Q-switched mode-locked pulses. Figure 6(a) shows the output pulse waveforms under the pump power of 1.1 W and the modulation signal of 100 kHz repetition rate, 60% duty cycle. The pulse has a Q-switched pulse envelope with 0.7 μs duration and 100 kHz repetition rate, and contains a train of ~30 mode-locked inner-pulses spaced at the laser cavity round-trip. There are also many noisy minor pulses filled in the spacing between the mode-locked inner-pulses. This phenomenon was also observed in Yb-doped FSF fibre laser^18^, as well as common Q-switched fibre lasers and gain-switched fibre lasers^33^,^34^. We believe this Q-switched mode locking regime is initiated by the combined effect of FSF and Q-switching^18^.

The mode-locking sub-pulses can be eliminated by decreasing the duty cycle of the modulation signal until the laser-on time is less than the building time of the mode-locked pulses. Then the pulse waveforms become smooth and stable, indicating a regular Q-switching regime. In this regime, the repetition rate follows the modulation signal, and the pulse width is related to the repetition rate, the duty cycle and the pump power. With the repetition rate fixed at 1 kHz and the pump power at 1.1 W, the shortest pulse width 35 ns can be obtained with the duty cycle set at 1%, as depicted in Fig. 6(b). In this case, the output power reached 36 mW. The pulse energy was 36 μJ, and the peak power was nearly 1 kW. The highest pulse energy 78 μJ can be obtained under the duty cycle of 50%, and the corresponding pulse width was 47 ns, and the peak power was 1.65 kW. Under the highest pump power of 1.1 W, with the flexible adjustment of the modulation repetition rate and duty cycle, the repetition rate of the Q-switched pulses can be adjusted in a wide range (from 1 kHz to >500 kHz) and the pulse duration can be tuned from 35 ns to 770 ns.

## Conclusion

In summary, we demonstrated the first versatile 2 μm pulses generation from Tm-based FSF lasers and provides a comprehensive report of the special behaviours of this laser. It was found that the implementation of FSF in Tm-doped fibre lasers enabled the distinct capacity of producing a rich variety of intriguing pulse dynamics, some of which have not been reported in Yb and Er based FSF lasers. Moreover, we greatly simplified the construction of FSF mode-locked fibre laser with an all-fibre integrated FBG-pair. The employment of an FBG pair can largely upgrade the practicability and the robustness of FSF mode locked lasers. Besides, unlike the commonly used scheme of combining FSF with complex NPR, we introduce an NOLM into an all-fibre FSF mode-locked laser, which successfully shortens the pulse duration from 24 ps to 8 ps. The unparalleled capacity of generating versatile pulses (including as short as 8 ps ultra-short pulses, pulse bundles, rectangular pulses, up to 78 μJ high energy nanosecond pulses and mode-locked Q-switched pulses) at the new operating wavelength in an ultra-compact format will largely expand the application range of the FSF mode locking techniques and provide a convenient and versatile alternative to the currently used techniques to generate on-demand 2 μm ps/ns pulses. A variety of applications can benefit from this compact 2 μm pulse generator, such as biomedicine, remote sensing, and pumping mid-infrared optical parametric oscillators. The intriguing pulse dynamics observed can also help contribute to the knowledge of mode-locked laser dynamics.

## Methods

A commercial autocorrelator based on 2nd harmonic generation was used to measure the pulse width of output pulses with maximum measurable pulse width of 80 ps. A short Tm-doped fibre amplifier was employed to amplify the peak power of the output pulses to reach the measurement criterion of the autocorrelator. The optical spectrum was measured by a 1200 nm–2400 nm optical spectrum analyser. The temporal characteristics were monitored with a 9 GHz InGaAs photodetector, a real-time oscilloscope (1.5 GHz bandwidth, 20 GSa/s sampling rate) and a 6 GHz radio frequency spectrum analyser.

## Additional Information

**How to cite this article**: Chen, H. *et al.* Diversified pulse generation from frequency shifted feedback Tm-doped fibre lasers. *Sci. Rep.* **6**, 26431; doi: 10.1038/srep26431 (2016).
